# Inflammation-Mediated Mechanisms of Arrhythmias After Acute Myocardial Infarction

**DOI:** 10.31083/RCM49761

**Published:** 2026-07-22

**Authors:** Huijie Guo, Jin Liu, Jinchun Wu

**Affiliations:** ^1^School of Clinical Medicine, Qinghai University, 810007 Xining, Qinghai, China; ^2^Department of Cardiovascular Medicine, Qinghai Provincial People’s Hospital, 810007 Xining, Qinghai, China

**Keywords:** acute myocardial infarction, arrhythmias, inflammation, systemic inflammatory response index, systemic immune–inflammation index

## Abstract

Acute myocardial infarction (AMI) remains a leading cause of cardiovascular death. Arrhythmias are the most common complication after AMI and can worsen disease progression and lead to sudden cardiac death (SCD). Therefore, prompt and accurate diagnosis, together with proactive and effective treatment, is crucial. This article reviews the mechanisms of inflammation in post-AMI arrhythmias, including inflammatory cell infiltration, cytokine release, oxidative stress, cardiac structural remodeling, and neuroimmune interactions. Additionally, this review discusses the predictive value of inflammatory markers and the clinical prospects of anti-inflammatory therapy, thereby providing a reference for future research and clinical prevention.

## 1. Introduction

Acute myocardial infarction (AMI) is one of the major cardiovascular diseases that cause disability and death worldwide. Although reperfusion therapy has significantly improved the prognosis of patients with AMI, arrhythmias remain an important cause of sudden cardiac death (SCD) as a common complication [1]. Available evidence suggests that the inflammatory response is not only involved in the initial pathological process of AMI, but also plays a key role in the occurrence and progression of arrhythmias after infarction. AMI is a pathological process in which coronary blood flow is markedly reduced or interrupted due to rupture of coronary atherosclerotic plaques, thrombosis, or coronary artery spasm, resulting in myocardial ischemia and necrosis. Particularly in East Asian populations, coronary artery spasm is one cause that cannot be ignored [2]. Inflammation, as the central pathological response after AMI, has a dual role: a moderate inflammatory response facilitates myocardial repair and defense via activation of the immune system, with inflammatory cells accumulating in the damaged area, removal of necrotic tissue, and promotion of fibrosis and cardiomyocyte repair [3]; alternatively, sustained inflammatory responses may aggravate infarct progression, disrupt the internal environment, and even trigger an inflammatory storm (cytokine storm, CS), leading to arrhythmias [4].

A study has shown that inflammation establishes an electrophysiological gradient between ischemic and non-ischemic regions, and that its associated mediators are both drivers of AMI and closely linked to the occurrence and progression of arrhythmias [5]. Arrhythmias are a common complication after AMI, and inflammation plays a key role in their pathogenesis. Early reperfusion strategies, including percutaneous coronary intervention (PCI) and thrombolytic therapy, have been shown to significantly reduce infarct size, attenuate inflammatory activation, and subsequently lower the incidence of early and late arrhythmias [6,7]. AMI complicated by arrhythmias poses a serious threat to the lives and health of patients. Therefore, in-depth exploration of the relationship between inflammation and post-AMI arrhythmias is of great significance for improving diagnostic accuracy, optimizing therapeutic strategies, and enhancing clinical management. This paper discusses the mechanisms of inflammation in post-AMI arrhythmias, analyzes the effects of different inflammatory pathways on cardiac electrophysiological properties, summarizes the current status and challenges of relevant clinical research, and provides a reference for future research directions and clinical intervention strategies.

## 2. Pathological Stages and Types of Arrhythmias in AMI

The pathological process of AMI can be divided into distinct stages, each with its own characteristics in the development of arrhythmias.

### 2.1 Acute Phase (Hours to Days): Ischemia-Dominated Electrical Disturbances

The acute phase of AMI is characterized by acute ischemia and necrosis of cardiomyocytes, and ischemia and hypoxia rapidly alter the electrophysiological properties of the myocardium: on the one hand, the stability of the cardiomyocyte membrane potential decreases, and the heterogeneity of action potential duration (APD) increases, forming an electrophysiological gradient between ischemic and non-ischemic regions [8]; alternatively, intracellular adenosine triphosphate (ATP) depletion leads to sodium–potassium pump dysfunction, which triggers potassium efflux and calcium overload, further exacerbating abnormalities in cell membrane depolarization [9]. The above changes provide an electrophysiological basis for reentrant arrhythmias, which clinically manifest as ventricular premature beats (VPBs), ventricular tachycardia (VT), and ventricular fibrillation (VF). These malignant ventricular arrhythmias (VAs) represent the main causes of acute SCD in patients with AMI. Notably, the extent of myocardial necrosis, often assessed by peak cardiac enzyme levels or infarct size on imaging, correlates strongly with the risk of malignant arrhythmias. Larger infarcts are associated with greater electrical heterogeneity, a higher inflammatory burden, and a higher incidence of VT/VF, as demonstrated in several clinical cohorts [10,11].

### 2.2 Subacute Phase (Days to Weeks): Inflammation-Driven Electrical Instability

In the subacute stage, the inflammatory response peaks, and inflammatory cells such as neutrophils and monocytes infiltrate the infarct area in large numbers, releasing proinflammatory factors including tumor necrosis factor-α (TNF-α), interleukin-1β (IL-1β), interleukin-6 (IL-6), and interleukin-17 (IL-17). During this period, tissue repair, extracellular matrix turnover, and early fibrosis become evident, providing a dynamic substrate for electrical instability [12]. Inflammatory mediators increase the risk of arrhythmias in two ways: first, the mediators directly modulate the expression and function of cardiomyocyte ion channels, leading to action potential abnormalities and slowed conduction; second, they impair intercellular electrical coupling through gap junction dysfunction.

TNF-α has been shown to reduce the peak sodium current (INa) by suppressing SCN5A transcription via nuclear factor kappa-light-chain-enhancer of activated B cells (NF-κB) activation and by promoting protein kinase C (PKC)-dependent phosphorylation of the Nav1.5 channel, which impairs its membrane trafficking and gating [13,14,15]. In addition to sodium channels, TNF-α suppresses multiple repolarizing potassium currents, including the transient outward potassium current (Ito) and the rapidly activating delayed rectifier potassium current (IKr), through downregulation of Kv4.2/Kv4.3 and hERG channel expression and through reactive oxygen species (ROS)-mediated channel dysfunction. TNF-α also disrupts intracellular calcium homeostasis by altering the open probability of the ryanodine receptor (RyR2) and inositol trisphosphate receptor signaling, thereby increasing diastolic calcium leak and delayed afterdepolarizations (DADs) [16,17].

Similarly, IL-1β directly modulates multiple ion channels: IL-1β reduces INa, potentially via SCN5A downregulation or post-translational modification; suppresses potassium currents (Ito, IKr) through ROS-mediated mechanisms; enhances L-type calcium current (ICaL), thereby prolonging action potential duration and promoting DADs [18,19]. IL-6, acting through Janus kinase (JAK)–signal transducer of activation (STAT) signaling, downregulates potassium channels (Kv4.3, hERG) and impairs IKs by affecting mitochondrial ATP production, while also increasing ICaL via the gp130/extracellular signal-regulated kinase (ERK) pathway [20,21]. IL-17 contributes to calcium-handling abnormalities by downregulating SERCA2a and may reduce Ito through NF-κB-dependent mechanisms [22]. These concerted actions of inflammatory cytokines create a highly arrhythmogenic substrate during the subacute phase. Thus, VAs remain elevated at this stage, and the risk of atrial arrhythmias, such as premature atrial contractions and paroxysmal atrial fibrillation (AF), increases. IL-6 and IL-17 are key cytokines in this phase. IL-6 promotes fibroblast activation and collagen deposition via STAT3 signaling, while IL-17 enhances neutrophil recruitment and ROS production [23,24]. The synergistic actions of IL-6 and IL-17 intensify electrical remodeling, calcium-handling abnormalities, and fibrotic changes, thereby creating a persistent arrhythmogenic substrate during the subacute post-infarction period.

Moreover, inflammatory cytokines can induce gap-junction dysfunction in cardiac myocytes by impairing connexin distribution, expression, and function. TNF-α and IL-1β downregulate connexin-43 (Cx43) expression and promote the lateralization of Cx43 away from intercalated discs, increasing conduction heterogeneity and forming a substrate for unidirectional block and reentry [25,26]. In addition, TNF-α differentially regulates connexin-40 (Cx40) expression through activation of the p38 mitogen-activated protein kinase (MAPK) signaling pathway, with region-specific effects that further exacerbate atrial and ventricular conduction dispersion [16,17]. A recent clinical translational study demonstrated that systemic inflammation induces rapid but reversible atrial electrical remodeling mediated by IL-6-dependent changes in Cx40 and Cx43 expression, providing direct clinical evidence linking inflammation to arrhythmia vulnerability [27].

### 2.3 Chronic Phase (More Than a Few Weeks): Arrhythmogenic Substrate Formation Mediated by Structural Remodeling

In the chronic stage, remodeling of cardiac structure becomes central, and collagen fibers gradually replace the infarcted area, forming fibrotic scars; compensatory hypertrophy occurs in surviving cardiomyocytes, and ventricular geometry changes (*e*.*g*., left ventricular dilatation). There are significant differences in conduction velocity between fibrotic scars and normal myocardial tissue, forming slow-conduction zones that provide a stable anatomical matrix for reentrant arrhythmias; concomitant fibrosis disrupts cardiomyocyte alignment, further exacerbating conduction heterogeneity [28]. Persistent VT, permanent AF, and atrioventricular block are the main clinical manifestations in this stage, with high recurrence rates of arrhythmias and poor prognosis. The characteristics of arrhythmias at different pathological stages are summarized in Table 1 (Ref. [29,30,31]).

**Table 1.** **Arrhythmia characteristics at different pathological stages of acute myocardial infarction (AMI)**.

Pathological staging	Time frame	Core pathological features	Mechanisms of arrhythmias	Common types of arrhythmias	Representative studies
Acute phase	0–72 hours	Acute ischemic necrosis of cardiomyocytes; membrane potential instability; calcium overload	Ischemia–non-ischemia electrophysiological gradient; reentry/triggered activity	Premature ventricular contractions, ventricular tachycardia, ventricular fibrillation	Ma J, Chen Q, et al. (2025) [29]
Subacute phase	3 days–2 weeks	Massive infiltration of inflammatory cells; release of inflammatory cytokines	Abnormal ion channel function; disruption of intercellular coupling	VAs, atrial premature contractions, paroxysmal AF	Nattel S, Maguy A, et al. (2007) [30]
Chronic phase	>2 weeks	Myocardial fibrosis; scar formation; cardiac structural remodeling	Formation of slow-conduction zones; increased conduction heterogeneity	Persistent ventricular tachycardia, permanent AF, atrioventricular block	Cofiño-Fabres C, Passier R, et al. (2023) [31]

AF, atrial fibrillation.

## 3. Types of Inflammation and Arrhythmias

### 3.1 Ventricular Arrhythmias (VAs)

The inflammatory response after AMI is closely related to VAs, and the incidence of VAs is significantly increased in patients with elevated inflammatory markers. Specifically, inflammatory factors such as TNF-α activate the PKC signaling pathway, leading to phosphorylation of the sodium channel Nav1.5 and a reduction in INa current density [32]. IL-1β modulates potassium ion channels, attenuates potassium currents, and causes abnormal action potential repolarization [33,34]. Alterations in the function of these ion channels increase myocardial electrical instability. Furthermore, IL-1β activates ROS through the NOD-like receptor thermal protein domain-associated protein 3 (NLRP3) inflammasome and inhibits sarco/endoplasmic reticulum Ca^2+^–ATPase 2a (SERCA2a) activity, resulting in diastolic calcium leakage and an increased amplitude of DADs, which facilitates triggered activity and ventricular arrhythmogenesis [35]. Inflammatory factors stimulate fibroblast proliferation and collagen deposition, leading to myocardial fibrosis, disruption of homogeneous electrical conduction, and the formation of anatomical reentry substrates. Abnormal electrophysiological properties, cardiomyocyte damage, and cardiac structural remodeling induced by inflammation can lead to severe VAs, including premature ventricular contractions, VT, and even VF [36]. A study has shown that high-sensitivity C-reactive protein (hs-CRP) levels are associated with the severity and prognosis in patients with VAs [37], which clinically manifests as VPBs, VT, and VF. Therefore, monitoring inflammatory markers is important for predicting the occurrence and progression of these arrhythmias. The electrophysiological consequences of inflammation are also reflected clinically as Corrected QT interval (QTc) interval prolongation, a recognized risk factor for Torsades de Pointes and SCD. Specifically, TNF-α and IL-6 reduce IKr density, whereas IL-1β impairs calcium handling, mechanisms that converge to prolong ventricular repolarization and increase vulnerability to polymorphic VT [38,39]. This integrated disruption of multiple ion channels underscores the complex arrhythmogenic substrate generated by inflammatory responses post-AMI.

In addition to the acute modulation of ion channels, inflammatory cytokines such as IL-6 and IL-17 contribute to ventricular electrical instability through distinct mechanisms. IL-6 activates the JAK–STAT pathway, leading to downregulation of potassium channels (*e*.*g*., IKr and the slowly activating delayed rectifier potassium current) and prolongation of APD, which predisposes patients to early afterdepolarizations and Torsades de Pointes [40]. IL-17 promotes oxidative stress and enhances fibroblast activity, contributing to fibrosis and conduction heterogeneity [41]. Both cytokines are associated with QTc prolongation, a key risk factor for lethal VAs and SCD.

Anti-inflammatory therapy has emerged as a new direction for the prevention of VAs, and a study has shown that anti-inflammatory drugs can reduce the risk of arrhythmias; however, excessive inhibition of inflammation may delay myocardial repair [42]. The specific efficacy and safety of these strategies still need to be verified in larger-scale clinical trials.

### 3.2 Atrial Arrhythmias (AAs)

The incidence of AF after AMI is high and associated with adverse clinical outcomes [43]. Among the contributing factors, inflammation plays a key role in the occurrence of AAs (such as AF, atrial flutter, and atrial tachycardia) after AMI. The incidence of AAs in patients with AMI is significantly elevated, especially AF, which is closely associated with increased levels of inflammatory markers (*e*.*g*., TNF-α and IL-6), and the inflammatory state may affect the success rate and prognosis of cardioversion in patients with AF [44]. Decreased left ventricular function after AMI leads to increased left atrial pressure, and atrial strain activates mechanosensitive ion channels and alters atrial electrophysiological characteristics; furthermore, the sympathetic nervous system and renin–angiotensin–aldosterone system are activated, promoting atrial electrical and structural remodeling. Inflammatory factors also induce atrial electrical and structural remodeling through the NF-κB and NLRP3 pathways, while atrial fibrosis and abnormal distribution of gap junction protein (Cx43) promote reentry formation [45,46,47]. In addition to Cx43, Cx40 is predominantly expressed in the atria and plays a crucial role in atrial conduction [48]. Downregulation of Cx40 by TNF-α and IL-1β disrupts atrial gap junction communication, facilitating reentry and increasing susceptibility to AF [49]. In addition, inflammation further exacerbates the risk of arrhythmias through mechanisms such as oxidative stress, abnormal calcium regulation, and fibrosis. Colchicine reduced the risk of post-AMI arrhythmias in the COLCOT trial, but did not improve the prognosis of VAs in the COPS trial; colchicine was effective when administered within 24 hours of AMI, whereas delayed administration was ineffective. The IL-1β inhibitor anakinra reduced AF recurrence in the VCU-ART3 trial but did not reduce the risk of VAs in the MATH-AMI trial [50]. In clinical practice, anti-inflammatory treatments (*e*.*g*., statins and colchicine) have shown potential to reduce the incidence of AAs in some studies, and therapies targeting specific inflammatory pathways (*e*.*g*., the IL-1β inhibitor anakinra) are also being explored. However, large-scale clinical trials are still needed for validation [51,52,53]. The specific mechanisms linking inflammation and AAs remain unclear and warrant further in-depth study to inform more effective prevention and treatment strategies in clinical practice. The main inflammatory mechanisms, their corresponding arrhythmia types, and potential therapeutic targets are summarized in Table 2.

**Table 2.** **Main inflammatory mechanisms, corresponding arrhythmia types, and potential therapeutic targets**.

Inflammatory mechanisms	Major inflammatory factors	Types of arrhythmias affected	Potential therapeutic targets
Abnormal ion channel function	TNF-α, IL-1β, IL-6, IL-17	VAs, AAs	Nav1.5 stabilizers, Kv-channel modulators, JAK–STAT inhibitors, p38 MAPK inhibitors
Calcium homeostasis imbalance	IL-1β, IL-18	VAs (triggered activity)	RyR2 stabilizers, SERCA2a agonists, NCX inhibitors
Myocardial fibrosis	TGF-β, IL-1β, IL-6, IL-17	VAs, AAs	Antifibrotic drugs (e.g., pirfenidone), TGF-β inhibitors, IL-6/IL-17 pathway inhibitors
Gap junction dysfunction	TNF-α, IL-1β, IL-6	AAs (especially AF), conduction block	Connexin-43/40 (Cx43/Cx40) modulators

TNF-α, tumor necrosis factor-α; IL-1β, interleukin-1β; IL-6, interleukin-6; IL-17, interleukin-17; TGF-β, transforming growth factor-β; VAs, ventricular arrhythmias; AAs, Atrial Arrhythmias; JAK–STAT, Janus kinase–signal transducer and activator of transcription; MAPK, mitogen-activated protein kinase; RyR2, ryanodine receptor; SERCA2a, sarco/endoplasmic reticulum Ca^2+^–ATPase 2a; NCX, sodium-calcium exchanger.

### 3.3 Sudden Cardiac Death (SCD)

SCD is defined as death from cardiac causes occurring within 1 hour of the onset of acute symptoms [54]. Studies have shown that inflammatory markers such as CRP and IL-6 are associated with the occurrence of SCD [55,56], and are subsequently of great value in predicting SCD. Post-AMI inflammation leads to ventricular electrical instability, thereby promoting malignant arrhythmias such as VF. Inflammation increases the risk of SCD by altering the function of ion channels and the distribution of gap junction protein (Cx43), increasing transventricular wall repolarization dispersion (transmural dispersion of repolarization (TDR) ≥40 ms), and creating a matrix for reentrant arrhythmias [57]. Meanwhile, inflammation exacerbates myocardial damage and apoptosis, leading to impaired cardiac pump function, and proinflammatory factors such as TNF-α, IL-1, and IL-6 can directly inhibit myocardial contractile function, thereby aggravating heart failure [58]. Currently, research data on inflammation and SCD remain limited, partly because patients with SCD die within a short period, making collecting relevant data difficult; meanwhile, survivors have CS in their bodies, so the correlation between inflammation and SCD needs to be further clarified.

### 3.4 Bradyarrhythmias

Inflammatory cytokines contribute to conduction disturbances by impairing gap junction function (Cx40/Cx43) and promoting fibrosis in the conduction system [59]. Inflammatory cytokines such as TNF-α, IL-1, and IL-6 are implicated in atrioventricular node disease [60]. IL-6 specifically downregulates Cx40 and Cx43, thereby impairing gap junction communication in the conduction system [61]. Additionally, reduced sodium current density in Purkinje fibers slows ventricular impulse propagation [62].

## 4. Mechanisms of Inflammation in Post-AMI Arrhythmias

Activated inflammatory cells secrete mediators such as TNF-α and IL-1β [63]. A study has found that elevated serum inflammatory marker levels in patients with AMI are closely associated with the occurrence of arrhythmias [44]. Elevated CRP levels not only reflect the degree of inflammatory response but also serve as an independent risk factor for predicting arrhythmias after AMI. Current research focuses on integrating cytokine profiles with traditional markers to enhance risk stratification.

### 4.1 Inflammatory Cells and Cytokines

After AMI, inflammatory cells such as neutrophils and macrophages infiltrate the infarcted area, releasing cytokines that cause myocardial damage and arrhythmias. Neutrophils are the first inflammatory cells to infiltrate the infarct zone and directly damage cardiomyocytes by releasing ROS and proteases, disrupting cell membrane integrity and affecting electrophysiological properties [64]. Macrophages are involved in the regulation of inflammatory responses and tissue repair. M1 macrophages secrete TNF-α, IL-1β, IL-6, and other factors that aggravate injury and electrical remodeling, whereas M2 macrophages regulate the formation of the arrhythmogenic matrix through anti-inflammatory and tissue repair functions [65]. A study has shown that inducing neutrophil apoptosis prevents CS and promotes the transformation of macrophages to the reparative M2 phenotype [66], thereby reducing myocardial damage. T and B lymphocytes participate in immunomodulation and inflammatory responses, regulate myocardial repair processes, and affect arrhythmogenic matrix formation [67]. IL-6 and IL-17 further amplify the inflammatory cascade. IL-6 stimulates Th17 differentiation and IL-17 production, thereby amplifying inflammation and fibrosis [68]. Elevated IL-17 levels correlate with an increased incidence of VT and AF post-AMI [69]. In addition, inflammatory factors can promote monocyte adhesion and migration, leading to microvascular occlusion, further aggravating myocardial ischemia, and indirectly causing arrhythmias [70]. Overall, myocardial damage and structural disruption induced by inflammatory cells can lead to abnormal electrical conduction in the heart. Although the risk of arrhythmias in patients with AMI can be assessed by monitoring inflammatory markers, standards and strategies for precise intervention remain inadequate. Inflammatory markers such as CRP, IL-6, TNF-α, and IL-1 have limited clinical validation as predictors of arrhythmias after AMI and require further evidence to establish unified standards.

### 4.2 Abnormal Ion Channel Function: The Molecular Basis of Electrophysiological Disorders

The normal function of ion channels in cardiomyocytes is essential for maintaining electrophysiological stability, and inflammatory factors interfere with ion channel expression and activity through transcriptional regulation and phosphorylation.

Sodium channels (Nav1.5, INa): TNF-α, IL-6, and IL-1β significantly impair cardiac sodium channel function. TNF-α and IL-6 inhibit *SCN5A* gene transcription by activating the NF-κB and JAK–STAT signaling pathways, thereby reducing Nav1.5 protein expression. Concurrently, PKC-mediated phosphorylation of Nav1.5 decreases the associated membrane localization and channel availability, resulting in a marked reduction in INa density. These changes slow phase 0 depolarization, reduce conduction velocity, and increase the likelihood of conduction block in the infarct border zone [33,71].

Potassium channels (Ito, IKr, IKs, IKur): TNF-α, IL-6, IL-1β, and IL-17 affect the expression and function of potassium channels. TNF-α suppresses multiple potassium currents, including Ito via reduced expression of Kv4.2/Kv4.3 channels mediated by inducible nitric oxide synthase induction, ROS generation, and Kv channel-interacting protein 2 (KChIP-2) inhibition; IKr via functional impairment of hERG channels through TNF-RI engagement and ROS production; IKs through sphingosine-1-phosphate generation and decreased cAMP; the ultra-rapid delayed rectifier potassium current (IKur) through reduced expression of the Kv1.5 potassium channel [72,73]. IL-6, through JAK–STAT signaling, reduces the expression of Kv4.3 (Ito) and HERG (IKr), prolonging APD and increasing transmural dispersion of repolarization [74]. IL-6 also impairs IKs by reducing mitochondrial ATP production, which is required for PKA-dependent phosphorylation of Kv7.1 potassium channel subunits [75]. Moreover, IL-17 decreases Ito through NF-κB-dependent downregulation of KChIP-2 [76]. Finally, IL-1β promotes ROS production by activating NLRP3 inflammasomes, and ROS-mediated oxidation modifies delayed-rectifier potassium channels, thereby inhibiting Ito and IKr, prolonging APD, and increasing transmural dispersion of repolarization, which predisposes to early post-depolarization [74].

Calcium channels (ICaL, SERCA2a, RyR2): Inflammatory cytokines also impair the expression and function of intracellular calcium channels, as well as the regulation of intracellular calcium homeostasis. IL-6 increases ICaL by enhancing Cav1.2 calcium channel function through the gp130/SHP2/ERK signaling pathway, a mechanism involving phosphorylation of the serine residue at position 1829 originally characterized for LIF. IL-18 increases calcium influx by activating the JAK–STAT pathway to regulate the expression of the L-type calcium channel (Cav1.2) and inhibiting SERCA2a activity, leading to intracellular calcium overload and triggering DADs, thereby providing a basis for triggered arrhythmias [77,78]. IL-1β also increases L-type calcium current (ICaL) through activation of cyclooxygenase and lipoxygenase pathways [74,79]. In addition, TNF-α enhances ICaL through NF-κB-dependent pathways, whereas IL-17 impairs calcium reuptake by downregulating SERCA2a [22].

### 4.3 Calcium Homeostasis Imbalance: A Key Trigger of Triggered Arrhythmias

Imbalance in intracellular calcium homeostasis is a central mechanism underlying triggered arrhythmias (*e*.*g*., polymorphic VT) after AMI, and inflammation disrupts calcium homeostasis through the following pathways.

Calcium leakage from the sarcoplasmic reticulum: After IL-1β activates NLRP3 inflammasomes, large amounts of ROS are produced and oxidatively modify sarcoplasmic RyR2, resulting in abnormal opening of the RyR2 channel and increased diastolic calcium leakage. Meanwhile, inflammatory factors inhibit SERCA2a activity, reduce sarcoplasmic reticulum calcium reuptake, and further aggravate intracellular calcium overload [80].

Mitochondrial calcium overload: Inflammation-mediated mitochondrial damage impairs the function of the mitochondrial calcium uniporter (MCU), leading to increased mitochondrial calcium uptake, decreased mitochondrial membrane potential, and increased ROS production, thereby forming a vicious cycle of “calcium overload–ROS production” that further disrupts calcium homeostasis [81]. Calcium overload induces DADs by activating the sodium-calcium exchanger (NCX) to generate inward current, which can trigger ectopic activity and arrhythmias when the DAD amplitude reaches threshold potential.

### 4.4 Cardiac Structural Remodeling: The Anatomical Basis of Reentrant Arrhythmias

Inflammation is an important driver of cardiac structural remodeling after AMI, providing an anatomical matrix for reentrant arrhythmias by inducing myocardial fibrosis, gap junction remodeling, and cardiomyocyte hypertrophy.

Myocardial fibrosis: Inflammatory factors such as transforming growth factor-β (TGF-β) and IL-1β stimulate fibroblast activation and conversion into myofibroblasts, which synthesize large amounts of type I and type III collagen, forming reparative fibrosis in the infarcted area and interstitial fibrosis in the non-infarcted area. Conduction velocity in fibrotic areas slows significantly (<0.3 m/s), creating a conduction barrier relative to normal myocardium (>0.5 m/s) and fulfilling the core condition for reentry, namely “slow conduction plus unidirectional block” [82].

Gap junction remodeling: Inflammatory factors downregulate Cx43 expression through the MAPKpathway. Meanwhile, these factors induce the redistribution of Cx43 from end-to-end junctions (intercalated discs) to the lateral cell borders between cardiomyocytes, disrupting the directionality and efficiency of electrical coupling and increasing conduction heterogeneity [57,83].

Myocardial hypertrophy: TNF-α and IL-6 induce compensatory hypertrophy of viable cardiomyocytes by activating the phosphatidylinositol 3-kinase–protein kinase B pathway, prolonging APD and causing imbalanced ion channel expression in hypertrophic cardiomyocytes, which further aggravates electrophysiological heterogeneity [84].

### 4.5 Oxidative Stress and Inflammation

Oxidative stress is an important pathophysiological process following AMI. In the infarcted area, myocardial ischemia and hypoxia increase intracellular ROS production, leading to an oxidative stress response [85]. A study has shown that oxidative stress can activate inflammatory signaling pathways, promote the release of inflammatory factors, and exacerbate inflammatory responses [86]. Consequently, the inflammatory response further aggravates oxidative stress, forming a vicious cycle. ROS can directly damage mitochondria, the endoplasmic reticulum, and other organelles in cardiomyocytes, impairing energy metabolism and electrophysiological properties and increasing the risk of arrhythmias. Oxidative stress is an important downstream effector of the inflammatory response, and antioxidant therapy may interrupt the inflammation–oxidative stress cycle by reducing ROS-mediated damage. However, clinical evidence for antioxidant therapy remains limited and requires validation in large-scale clinical trials.

### 4.6 Neuroimmune Interaction: An Important Regulator of Autonomic Nervous System Imbalance

The post-AMI inflammatory response can disrupt autonomic balance through central and peripheral pathways, exacerbating the risk of arrhythmias. Peripheral inflammatory factors (such as IL-1β) activate microglia in the paraventricular nucleus (PVN) through the blood–brain barrier or vagal afferent pathways, and activated microglia release IL-1β and TNF-α, further stimulating sympathetic nerve centers and increasing sympathetic activity [87,88]. Meanwhile, inflammation-mediated myocardial injury can induce abnormal hyperplasia of sympathetic nerve endings (nerve sprouting), and sympathetic nerve density in the peri-infarct region increases significantly. In contrast, vagal nerve density decreases, creating an imbalance characterized by “sympathetic overactivation + vagus nerve inhibition” [89]. Sympathetic excitation increases calcium influx and APD dispersion via β1 receptors, whereas vagal nerve inhibition weakens the protective antiarrhythmic effects of vagal activity, thereby jointly aggravating electrical instability. PVN neurons, under optogenetic control, can inhibit sympathetic hyperactivation [90]. Therefore, suppressing sympathetic overexcitation and maintaining cardiac autonomic balance may present a new approach to treating arrhythmias after AMI.

### 4.7 Cell Death and the Inflammatory Cycle: An Important Link in Inflammatory Amplification

Apoptosis and pyroptosis are important forms of cell death after AMI, and both are closely related to the inflammatory response. Apoptosis is a programmed form of cell death in which apoptotic cardiomyocytes release IL-1α and RNA after AMI, thereby activating an inflammatory response [91]. Pyroptosis is a gasdermin D (GSDMD)-mediated inflammatory form of cell death involving membrane pore formation. After AMI, cardiomyocyte death (apoptosis and pyroptosis) forms a negative feedback cycle with inflammatory responses, further aggravating the risk of arrhythmias.

Ischemia, hypoxia, and inflammatory factors induce apoptosis in cardiomyocytes, and apoptotic cells release damage-associated molecular patterns (DAMPs), such as high mobility group box 1 (HMGB1) and ATP. These DAMPs bind to TLR4 receptors on the surface of macrophages, activate the NF-κB pathway, promote the release of proinflammatory factors, and amplify the inflammatory response. Pyroptosis involves activation of the NLRP3 inflammasome and cleavage of caspase-1, which in turn cleaves GSDMD to form membrane pores, resulting in cell swelling and rupture and releasing potent proinflammatory factors such as IL-1β and IL-18, thereby exacerbating the inflammatory cascade [92,93]. This feedback loop of inflammation and cell death enlarges the area of myocardial damage and further aggravates electrophysiological and structural abnormalities. A previous study has found that stem cell therapy can promote myocardial repair and reduce inflammatory responses by differentiating into cardiomyocytes and secreting cytokines, thereby reducing the risk of arrhythmias [94]. These studies provide new ideas and methods for the clinical treatment of arrhythmias after AMI, but further clinical trials are needed to verify their safety and efficacy. The core mechanisms, key effects, and arrhythmogenic consequences of inflammation in post-AMI arrhythmias are summarized in Table 3.

**Table 3.** **Main mechanisms of inflammation in post-AMI arrhythmias**.

Core mechanism	Key effects	Consequences
Initiation of inflammatory cell infiltration	Neutrophils, macrophages, and other inflammatory cells infiltrate the tissue and release IL‑1β, TNF‑α, and IL‑6	Initiates and amplifies the inflammatory response, forming the basis of all downstream mechanisms
Electrophysiological remodeling	Ion channel dysfunction; slowed conduction; prolonged and heterogeneous repolarization (APD/TDR↑, QTc↑) mediated by TNF-α, IL-6, IL-17	Creates a substrate for reentry and triggered activity; increases the risk of Torsades de Pointes and polymorphic VT
Structural remodeling	Inflammatory factors lead to myocardial fibrosis and remodeling of gap junctions (Cx43, Cx40)	Forms an anatomical reentry matrix, causing conduction block
Calcium homeostasis imbalance	Inflammatory factors and ROS cause calcium leakage from the sarcoplasmic reticulum and intracellular calcium overload	Induces delayed depolarization, resulting in triggered arrhythmias
Neuroimmune dysregulation	Inflammatory signaling centers are activated, leading to sympathetic excitation and vagal inhibition	Significantly increases myocardial electrical instability and lowers the arrhythmia threshold
Gap junction dysfunction	Inflammatory factors directly downregulate Cx43 and Cx40, impairing intercellular electrical coupling	Causes acute conduction slowing, promoting reentry and conduction block

APD, action potential duration; TDR, transmural dispersion of repolarization; QTc, Corrected QT interval; ROS, reactive oxygen species; VT, ventricular tachycardia. The up arrow (↑) consistently signifies that the parameter is elevated, prolonged, or increased relative to the normal/baseline level.

## 5. Clinical Significance of Inflammatory Markers

Inflammatory markers can quantify the inflammatory status of patients with AMI, thereby providing an objective basis for arrhythmia risk stratification and prognostic assessment.

### 5.1 Traditional Inflammatory Markers: Widely Used in Clinical Practice but With Limited Specificity

C-reactive protein (CRP): CRP is an acute-phase protein synthesized by the liver. CRP levels begin to increase 6–12 hours after AMI and peak at 48–72 hours. Meanwhile, hs-CRP levels are significantly associated with the incidence of VAs after AMI. A previous study has shown that patients with AMI and hs-CRP >10.3 mg/L have an increased risk of malignant VAs during hospitalization [95]. 

White blood cell count (WBC): WBC count, especially the neutrophil count, rises rapidly after AMI and reflects the degree of inflammatory cell infiltration. A previous study has found that elevated WBC count is an independent predictor of malignant VAs during hospitalization in patients with a first AMI [96].

Procalcitonin (PCT): PCT is a sensitive marker of bacterial infection. However, a recent study has found that increased PCT levels after AMI are associated with greater inflammation severity and a higher risk of arrhythmias [97]. 

### 5.2 Novel Inflammatory Composite Indicators: Higher Predictive Value

Traditional single markers cannot fully capture the complex interactions within the inflammation–immunity–coagulation system. Thus, by integrating multidimensional parameters, novel composite indices significantly improve predictive performance.

Systemic inflammatory response index (SIRI): SIRI is calculated based on peripheral blood neutrophil (N), monocyte (M), and lymphocyte (L) counts (SIRI = N × M/L) and is used to assess the intensity of the inflammatory response [98]. Neutrophil and monocyte infiltration is closely associated with the release of inflammatory mediators that alter the electrophysiological properties of cardiomyocytes and increase the risk of arrhythmias. SIRI has been used to assess prognosis in a variety of diseases, including acute coronary syndrome and ischemic stroke [99,100]. A study has found that SIRI is closely related to the prognosis of patients with AMI [101], showing potential value in assessing the risk of arrhythmias in this population. Indeed, one study found that SIRI alone had good but suboptimal predictive accuracy for post-infarction AF (area under the curve (AUC) = 0.813 vs. 0.868 for the combined model), suggesting that this metric should be combined with traditional clinical factors [102].

Although SIRI has shown good prognostic value across a range of diseases, its application in post-AMI arrhythmias still faces challenges. Currently, most of the existing evidence regarding the use of SIRI to predict arrhythmias comes from retrospective studies, and the independent predictive value of SIRI requires further verification in prospective cohorts.

Systemic immune–inflammation index (SII): The SII is calculated from peripheral blood neutrophil, lymphocyte, and platelet (P) counts (SII = N × P/L) and reflects the degree of inflammatory response as well as the balance between immunity and coagulation [103]. High SII levels are an independent predictor of poor prognosis in patients with AMI and may serve as a potential predictor of arrhythmias [104]. Currently, SII is widely used for prognostic assessment across various diseases, particularly tumors and cardiovascular diseases. However, the use of the SII in post-AMI arrhythmias remains limited, and more prospective studies are needed to confirm its predictive value.

Neutrophil-to-lymphocyte ratio (NLR): Elevated NLR reflects an enhanced neutrophil-driven inflammatory response and lymphocyte-mediated immunosuppressive state. In AMI patients with NLR >5, the risk of VT/VF is increased within 30 days, making NLR a simple and practical indicator for clinical risk assessment [105]. A previous study has demonstrated that NLR >5 is independently associated with an approximately 2-fold increased risk of adverse events, including cardiovascular death, all-cause mortality, and thromboembolic events, in patients with AF [106]. The underlying mechanism may involve neutrophil-driven atrial fibrosis via the IL-6/IL-17/TGF-β signaling pathway and enhanced oxidative stress.

### 5.3 Cardiac Enzyme Markers and Arrhythmia Prediction

Cardiac troponin (TnT/TnI), creatine kinase (CK), and creatine kinase-MB (CK-MB) are routinely measured in AMI for diagnostic and prognostic purposes. Elevated levels reflect the extent of myocardial necrosis and have been associated with an increased risk of arrhythmias. Peak TnT levels correlate with infarct size and predict both early VAs and late AF [107,108]. Similarly, high CK-MB levels are associated with electrical instability and may serve as an indirect marker of the arrhythmogenic substrate. However, these markers lack specificity for inflammatory activity and primarily indicate myocardial injury rather than arrhythmogenic mechanisms per se.

### 5.4 Cytokine Biomarkers: TNF-α, IL-1, and IL-6

**Fig. 1.**
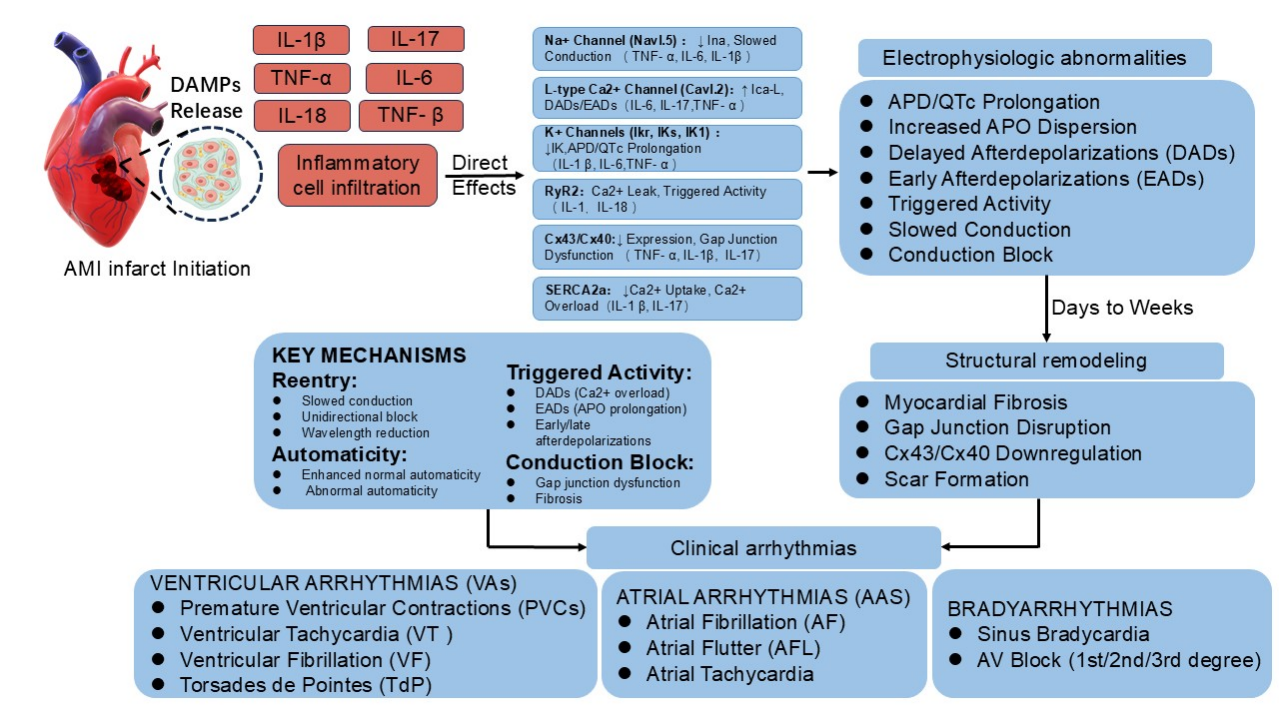
**Mechanism of arrhythmias mediated by inflammation after acute myocardial infarction (AMI)**.

Specific inflammatory cytokines, such as TNF-α, IL-1β, and IL-6, are increasingly recognized for their prognostic value in post-AMI arrhythmias. TNF-α levels correlate with ventricular electrical remodeling and predict the occurrence of VT [109,110]. IL-1β is implicated in calcium-handling disturbances and has been linked to both AAs and VAs; elevated post-AMI IL-1β levels are associated with a higher incidence of AF [111]. IL-6 reflects systemic inflammation and independently predicts SCD and malignant arrhythmias in patients with AMI [59]. Despite their pathophysiological relevance, routine clinical use of these cytokines remains limited due to assay variability, cost, and the lack of standardized cut-off values. Current research focuses on integrating cytokine profiles with traditional markers to enhance risk stratification. The integrated mechanisms of inflammation-mediated arrhythmias after AMI are illustrated in Fig. 1.

## 6. Anti-inflammatory Therapies: Current Evidence and Future Directions

Current evidence supporting anti-inflammatory therapies for post-AMI arrhythmias remains limited. The CANTOS trial demonstrated that canakinumab (an IL-1β inhibitor) reduced recurrent cardiovascular events but did not specifically assess arrhythmic outcomes. The COLCOT trial showed that colchicine reduced the risk of ischemic events; however, the effect of colchicine on VAs was not significant, a finding corroborated by recent real-world analyses [112]. In contrast, the VCU-ART3 trial demonstrated that anakinra, an IL-1 receptor antagonist, significantly reduced heart failure and inflammatory markers following STEMI [113], suggesting potential benefits for arrhythmia prevention by attenuating ventricular remodeling; however, dedicated arrhythmic endpoints were not evaluated. These findings highlight the need for targeted trials to evaluate anti-cytokine therapies for arrhythmia prevention post-AMI.

## 7. Limitations

First, the current evidence is derived largely from observational and preclinical studies, with a scarcity of large-scale randomized controlled trials. Second, the predictive utility of novel inflammatory indices (*e*.*g*., SIRI and SII) requires further validation in prospective cohorts. Finally, the long-term safety and optimal application of anti-inflammatory therapies in patients with AMI remain to be clarified. Future research should focus on addressing these gaps.

## 8. Outlook

Inflammation contributes to the onset and progression of arrhythmias after AMI through multiple pathways, with significant interindividual variability in the intensity and activation patterns of inflammatory responses. Future research should prioritize the following: elucidating the mechanisms of specific inflammatory pathways in arrhythmogenesis; developing highly sensitive and specific inflammatory biomarkers, supported by artificial intelligence-integrated multimodal analysis, to improve risk stratification; exploring multi-target anti-inflammatory therapeutic strategies; conducting well-designed large-scale clinical trials to evaluate the efficacy and safety of anti-inflammatory interventions; establishing individualized treatment plans based on inflammatory profiling. Importantly, the implantation of cardiac rhythm devices remains an effective means of preventing arrhythmia-related mortality post-AMI, underscoring the need for precise patient stratification and tailored therapy. Indeed, by advancing our understanding of inflammatory mechanisms and translating these insights into targeted prevention and treatment strategies, including both pharmacological and device-based approaches, the prognosis of patients with AMI may be further improved.

## 9. Conclusions

In summary, inflammation plays a pivotal role in the pathogenesis of arrhythmias following AMI, contributing to electrical instability, structural remodeling, and autonomic imbalance through multiple interconnected pathways. While inflammatory markers such as CRP, SIRI, SII, and inflammatory cytokines show promise for risk stratification, their clinical utility requires further validation in prospective studies. Anti-inflammatory therapies, including colchicine and IL-1β inhibitors, have shown potential for reducing arrhythmic events; however, the efficacy and safety profiles of these approaches require confirmation in larger randomized trials. Future research should focus on elucidating specific inflammatory pathways, developing targeted therapeutic strategies, and establishing individualized treatment protocols based on inflammatory status. Ultimately, a deeper understanding of inflammation-arrhythmia interactions may pave the way for improved prognostic assessment and therapeutic interventions in patients with AMI.
